# SppI Forms a Membrane Protein Complex with SppA and Inhibits Its Protease Activity in Bacillus subtilis

**DOI:** 10.1128/mSphere.00724-20

**Published:** 2020-10-07

**Authors:** Gabriela Henriques, Stephen McGovern, Jolanda Neef, Minia Antelo-Varela, Friedrich Götz, Andreas Otto, Dörte Becher, Jan Maarten van Dijl, Matthieu Jules, Olivier Delumeau

**Affiliations:** a Université Paris-Saclay, INRAE, AgroParisTech, Micalis Institute, Jouy-en-Josas, France; b University of Groningen, University Medical Center Groningen, Department of Medical Microbiology, Groningen, The Netherlands; c University of Greifswald, Centre of Functional Genomics of Microbes, Institute of Microbiology, Department of Microbial Proteomics, Greifswald, Germany; d Department of Microbial Genetics, University of Tübingen, Tübingen, Germany; University of Iowa

**Keywords:** *Bacillus subtilis*, signal peptide peptidase, lantibiotic resistance, membrane protein complex, enzymatic regulation, proteases

## Abstract

The membrane protease SppA of Bacillus subtilis was first described as a signal peptide peptidase and later shown to confer resistance to lantibiotics. Here, we report that SppA forms octameric complexes with YteJ, a membrane protein of thus-far-unknown function. Interestingly, *sppA* and *yteJ* deletion mutants exhibited no protein secretion defects. However, these mutant strains differed significantly in their resistance to antimicrobial peptides. In particular, *sppA* mutant cells displayed increased sensitivity to the lantibiotics nisin and subtilin and the human lysozyme-derived cationic antimicrobial peptide LP9. Importantly, YteJ was shown to antagonize SppA activity both *in vivo* and *in vitro*, and this SppA-inhibitory activity involved the C-terminal domain of YteJ, which was therefore renamed SppI. Most likely, SppI-mediated control is needed to protect B. subtilis against the potentially detrimental protease activity of SppA since a mutant overexpressing *sppA* by itself displayed defects in cell division. Altogether, we conclude that the SppA-SppI complex of B. subtilis has a major role in protection against antimicrobial peptides.

**IMPORTANCE** Our study presents new insights into the molecular mechanism that regulates the activity of SppA, a widely conserved bacterial membrane protease. We show that the membrane proteins SppA and SppI form a complex in the Gram-positive model bacterium B. subtilis and that SppI inhibits SppA protease activity *in vitro* and *in vivo*. Furthermore, we demonstrate that the C-terminal domain of SppI is involved in SppA inhibition. Since SppA, through its protease activity, contributes directly to resistance to lantibiotic peptides and cationic antibacterial peptides, we propose that the conserved SppA-SppI complex could play a major role in the evasion of bactericidal peptides, including those produced as part of human innate immune defenses.

## Abstract (precis)

Our study presents new insights into the molecular mechanism that regulates the activity of SppA, a widely conserved bacterial membrane protease. We show that the membrane proteins SppA and SppI form a complex in the Gram-positive model bacterium B. subtilis and that SppI inhibits SppA protease activity *in vitro* and *in vivo*. Furthermore, we demonstrate that the C-terminal domain of SppI is involved in SppA inhibition. Since SppA, through its protease activity, contributes directly to resistance to lantibiotic peptides and cationic antibacterial peptides, we propose that the conserved SppA-SppI complex could play a major role in the evasion of bactericidal peptides, including those produced as part of human innate immune defenses.

## INTRODUCTION

Bacterial proteases play important physiological roles that range from digesting partially damaged proteins to degrading a wide range of regulatory factors, and they are involved in many cellular pathways inside and outside the cell. For example, in the cytoplasm, the Clp complexes are of major importance for the quality control of proteins, and they are responsible for the degradation of misfolded proteins that are inevitably produced during heat stress or stationary phase (1) (for a review, see references 2 and 3). Particular proteases can serve multiple roles. For instance, outside the cell, secreted proteases may act as “feeding proteases” by digesting proteins and providing the cell with peptides and amino acids. In Bacillus subtilis, the two major feeding exoproteases NprE and AprE have also been shown to reduce autolytic activity (4). Moreover, AprE is a regulator of cellular processes, such as competence, as it is involved in the production of the quorum-sensing peptide competence and sporulation factor (CSF) (5).

In the protein secretion pathway, proteases are crucial members that act sequentially to allow the secretion of a fully functional protein. First, to release the premature secretory protein after its translocation through the translocon, a signal peptidase cleaves the signal peptide (SP) at the extracytoplasmic surface of the membrane, leaving the SP inserted in the membrane (6, 7). Second, a quality control mechanism, based on proteins belonging to the HtrA family of proteases, assists in folding or degrades misfolded secretory proteins at the *trans* side of the membrane. B. subtilis HtrA and HtrB are membrane-bound members of this protein family, which are overexpressed in response to heat and secretion stress conditions that induce protein misfolding or aggregation (8, 9). Finally, the remaining SPs in the membrane are cleaved by an intramembrane protease member of the S2P protease family, such as RseP in Escherichia coli or RasP in B. subtilis (10).

In B. subtilis, another membrane protease, named SppA, has been annotated as a signal peptide peptidase (11). Indeed, E. coli SppA (12) and B. subtilis SppA share 49% identical or conserved residues over their C-terminal domains, and the maturation of an overexpressed and secreted α-amylase (AmyQ) protein was slower in a Δ*sppA* mutant strain (11). However, by specifically addressing SP cleavage *in vivo*, Saito et al. provided evidence that RasP could be the main SP peptidase in B. subtilis (10), suggesting that SppA has a secondary or condition-dependent role in degrading SPs. Kingston et al. showed that *sppA* gene expression was upregulated after nisin stress in B. subtilis, consistent with this gene being a member of the cell envelope stress-responsive σ^W^ regulon (13). Interestingly, as the deletion of *sppA* negatively affected resistance to nisin, these authors assigned a new role to SppA in B. subtilis resistance to lantibiotic peptides, possibly through its protease activity.

The present study was aimed at understanding the role of the protein of unknown function YteJ. Briefly, we show by blue native PAGE (BN-PAGE) and tandem affinity purification (TAP) that SppA interacts with YteJ, implying that these two membrane proteins form a complex. Furthermore, by biochemical and genetic analyses, we show that YteJ, here renamed SppI, is an inhibitor of SppA’s proteolytic activity and that the C-terminal domain of SppI has a crucial role in the regulation of SppA activity. Finally, we demonstrate that SppA is involved in bacterial resistance to lantibiotic and cationic antibacterial peptides.

## RESULTS

### SppA and SppI form a membrane protein complex in B. subtilis.

**FIG 1:**
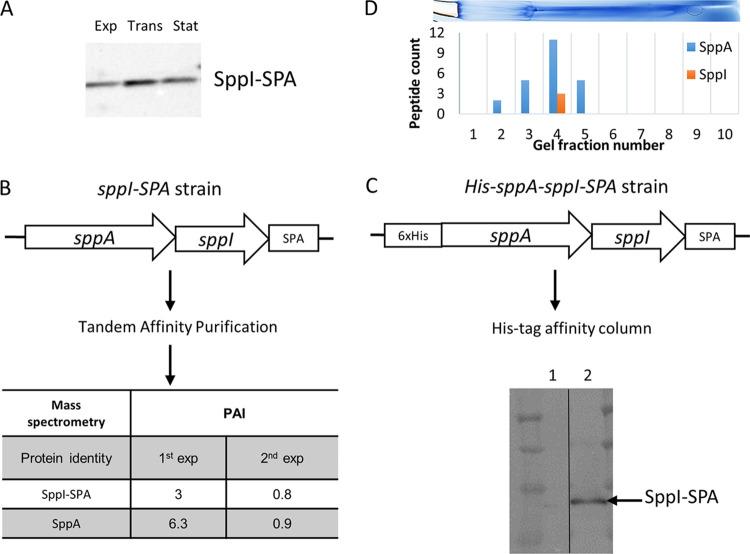
(A) Cell samples of a strain expressing SppI-SPA were taken during exponential (Exp), transition (Trans), and stationary (Stat) phases of growth. The membrane fractions were obtained after ultracentrifugation, and the sample volumes were adjusted to the OD at 600 nm measured before loading on SDS-PAGE gels. Anti-FLAG antibodies were used to reveal SppI-SPA. (B) Two liters of LB medium was inoculated with cells expressing SppI-SPA, and cells were harvested at the mid-exponential phase of growth. Tandem affinity purification from the membrane fractions obtained after ultracentrifugation was performed. The results of two independent experiments are shown as PAI values. (C) His tag purification of His-SppA. After elution, the proteins were separated by SDS-PAGE, and a Western blot using commercial anti-FLAG for detecting SppI-SPA is shown. Lane 1, proteins eluted after the His tag purification procedure from an SppI-SPA-expressing strain, used as a control; lane 2, proteins eluted after the His tag purification procedure from a 6×His-SppA-SppI-SPA-expressing strain. (D) BN-PAGE of the membrane fraction of B. subtilis 168 (BSB1). The gel was cut into 10 fractions, as indicated on the gel, and each fraction was analyzed by MS. Results are shown as spectral counts for SppA and SppI detected in each fraction.

The two genes *sppA* and *sppI* are organized in an operon (14), suggesting the possibility of an interaction between the two proteins. Strains of B. subtilis expressing a C-terminal fusion of both proteins SppA and SppI with the SPA tag sequence were constructed as described previously (15). Western blots using commercial antibodies against the FLAG tag, part of the sequential peptide affinity (SPA) tag, were first used to check the levels of expression of the two SPA-tagged proteins during the growth of B. subtilis. SppI-SPA was detected by Western blotting in the exponential, transition, and stationary growth phases (Fig. 1A) and estimated to be produced five times less than SecDF but twice more than SecG (see Fig. S1 in the supplemental material). On the contrary, although the DNA sequence of the *sppA*-SPA fusion was verified to be correct, SppA-SPA was not detected by Western blotting (Fig. S1). As the structure of the active form of SppA has revealed that the C-terminal domain is subject to removal by self-cleavage (16), we assumed that the C-terminal tag might have been degraded. Since SppI is predicted to be a membrane protein with at least two transmembrane domains, possibly three, we used a protocol adapted to membrane proteins as described previously (17) for tandem purification of potential SppI protein partners by pulldown with SppI-SPA. The tandem-affinity-purified proteins along with SppI-SPA were analyzed by liquid chromatography-tandem mass spectrometry (LC-MS/MS). In two independent experiments, SppA was identified as the major protein copurified with SppI-SPA (Fig. 1B). This strongly indicated that the two proteins form a membrane protein complex. The SppI-SPA and SppA ratios in the complex were estimated by calculating their protein abundance index (PAI) values as described previously (18). The PAI values obtained from two independent experiments were 0.8 (SppI) and 0.9 (SppA) in one experiment and 3 (SppI) and 6.3 (SppA) in the second one. The ratio of these values suggested that the SppA-SppI complex stoichiometry could range from 1:1 to 1:2. Knowing that the *sppA* and *sppI* genes form an operon, a 1:1 ratio is plausible.

10.1128/mSphere.00724-20.2 FIG S1 Western blot analysis of the expression levels of SecDF-SPA, SecG-SPA, SppA-SPA, and SppI-SPA in the corresponding strains. Three samples of cells of each strain were taken during the exponential phase (lane 1), transition phase (lane 2), and stationary phase (lane 3). After centrifugation, a constant ratio of the OD/volume of buffer was used to resuspend the cells of all samples. Following sonication and centrifugation, equal volumes of supernatants for each sample were loaded on SDS-PAGE gels, and Western blotting was performed. The arrows indicate the tagged proteins detected and are positioned to indicate the full size expected. The quantities were estimated by the band intensities of the SPA-tagged proteins using Image Lab software (Bio-Rad). Degradation products could be observed for SecDF-SPA. Download FIG S1, TIF file, 0.7 MB. Copyright © 2020 Henriques et al. 2020 Henriques et al. This content is distributed under the terms of the Creative Commons Attribution 4.0 International license.

To verify the observed protein interaction between SppA and SppI, we constructed a B. subtilis strain in which sequences encoding a 6×His tag and the SPA tag were fused to the 5′ and 3′ ends of the *sppA sppI* operon, respectively (Fig. 1C). The *6×his*-*sppA sppI-spa* DNA construct was ligated to the isopropyl β-d-1-thiogalactopyranoside (IPTG)-inducible P_hyperspank_ (P*_hs_*) promoter in plasmid pDR111 and, upon the transformation of the BSB1 strain, integrated into the *amyE* locus by a double recombination event. This construct allowed the simultaneous expression of the two tagged proteins 6×His-SppA and SppI-SPA in B. subtilis upon induction by IPTG. After collecting the induced cells, the cytosolic membrane fractions were ultracentrifuged and solubilized with 1% *n*-dodecyl β-d-maltoside (DDM)-containing buffer. The membrane fractions were incubated with Ni-nitrilotriacetic acid (NTA) affinity resin to capture 6×His-SppA. A control experiment was performed under the same conditions with B. subtilis strain GHBs018 that expressed only SppI-SPA. After extensive washes of the columns, the eluted proteins were separated by SDS-PAGE. Western blotting using anti-FLAG antibodies revealed that SppI-SPA was present in the eluate fraction of the samples from the 6×His-SppA- and SppI-SPA-expressing strain, while no SppI-SPA was detectable for the control strain (Fig. 1C). We conclude that both the copurification of SppA with SppI-SPA and the reciprocal copurification of SppI-SPA with 6×His-SppA imply that SppA and SppI are present as a complex in the B. subtilis membrane.

### An ∼480-kDa SppA-SppI complex in B. subtilis.

To estimate the SppA-SppI complex molecular weight (MW) and stoichiometry, we used BN-PAGE coupled with MS. After electrophoresis, the detected gel band was cut into 10 slices, and proteins in each slice were identified by LC-MS/MS. The spectral counts associated with SppA and SppI were used to approximate the quantities of the two proteins present in each gel slice. The SppA protein was detected in slices 2 to 5, with a maximum spectral number in slice 4, whereas SppI-specific peptides were detected by LC-MS/MS only in slice 4 (Fig. 1D). The highest quantities of SppA and SppI found in slice 4 corresponded to the position of the 480-kDa MW marker. Since Nam et al. reported that SppA (36.6 kDa) is present as an octamer (19), we expect the SppA subcomplex within the SppA-SppI complex to be 292 kDa (8 × 36.6 kDa). Hence, as the most probable SppA/SppI ratio would be 1:1 (our above-mentioned results), it seems plausible that SppI (19.2 kDa) also forms an octameric subcomplex within the SppA-SppI complex with a theoretical MW of 153 kDa (8 × 19.2 kDa). The theoretical MW for an 8×SppA/8×SppI complex is therefore expected to be 445 kDa, which is in agreement with our estimation of 480 kDa based on BN-PAGE. Subsequently, gel filtration profiles obtained during individual protein or protein complex purifications confirmed this MW estimation (Fig. S2). We therefore conclude that SppA and SppI form a membrane-associated protein complex in B. subtilis, which is composed of eight SppA and eight SppI subunits.

10.1128/mSphere.00724-20.3 FIG S2 Gel filtration chromatography of the affinity-purified His-tagged proteins His-SppA^K199A^, His-SppA, His-SppA-SppI, and His-SppI. (A) Elution profiles from a Superdex 200 GL 10/300 gel filtration column. The elution volumes of the MW markers and their sizes are indicated with triangles at the top. (B) Collected fractions analyzed by SDS-PAGE. Download FIG S2, TIF file, 2.8 MB. Copyright © 2020 Henriques et al. 2020 Henriques et al. This content is distributed under the terms of the Creative Commons Attribution 4.0 International license.

### The SppA-SppI complex is dispensable for protein secretion.

SppA was initially described as a member of the secretion apparatus since an *sppA* deletion was observed to slow down the maturation of the hypersecreted α-amylase AmyQ (11). To investigate the possible involvement of SppI in protein secretion, we first assessed the effect of the deletion of *sppI* or the entire *sppA sppI* operon on the secretion of another α-amylase, AmyM. However, *sppI* or *sppA sppI* mutant cells in the exponential or early stationary growth phases did not show any differences in the secretion of AmyM into the growth medium, and the same was true for the *sppA* single mutant control strain (Fig. S3A). We also tested the effects of the overexpression of *sppA*, *sppI*, and the *sppA sppI* operon on AmyM secretion. Upon *sppI* and *sppA sppI* overexpression, the levels of secreted AmyM were very similar, while the overexpression of *sppA* resulted in a different profile of proteins in the medium as shown by SDS-PAGE, probably due to cell lysis. Notably, the *sppA*-overexpressing cells secreted drastically reduced amounts of AmyM, in contrast to what was recently described for *sppA* overexpression in Bacillus licheniformis (20). Therefore, we verified whether the maturation kinetics of AmyQ were affected by the deletion of *sppI* and/or *sppA* or by the overexpression of *sppA* and *sppI.* As shown by pulse-chase labeling experiments to monitor the processing of [^35^S]methionine-labeled pre-AmyQ, neither the deletion nor the overexpression of *sppA* and/or *sppI* influenced the maturation kinetics of AmyQ (Fig. S3B). Taken together, these results show that if SppA plays a role in protein secretion, it is very limited or condition dependent. This is also true for SppI, as although SppI forms a complex with SppA, no effects on α-amylase secretion levels or maturation kinetics were observed in *sppI*-deleted or -overexpressing strains.

10.1128/mSphere.00724-20.4 FIG S3 (A) Effect of deletion or overexpression of *sppA*, *sppI*, and *sppA-sppI* on secretion levels of AmyM. All strains were transformed with plasmid pCS73 (expressing the *amyM* gene under the control of a constitutive promoter) except for the WT strain (BSB1) transformed with pCS74 (same as pCS73 without the *amyM* gene) used as a control (lane 1). Cells were taken in the early stationary phase of growth, and proteins from the medium were TCA precipitated and analyzed by SDS-PAGE. Lane 1, BSB1(pCS74) (empty plasmid); lane 2, BSB1(pCS73); lane 3, Δ*sppA* (pCS73); lane 4, Δ*sppI*(pCS73); lane 5, P*_hs_sppA*(pCS73); lane 6, P*_hs_sppI*(pCS73); lane 7, P*_hs_sppA sppI*(pCS73). (B) Effect of the deletion or overexpression of *sppA*, *sppI*, and the operon *sppA sppI* on the processing of pre-AmyQ. Download FIG S3, TIF file, 2.8 MB. Copyright © 2020 Henriques et al. 2020 Henriques et al. This content is distributed under the terms of the Creative Commons Attribution 4.0 International license.

### SppA is needed for long-term survival of B. subtilis cells.

**FIG 2:**
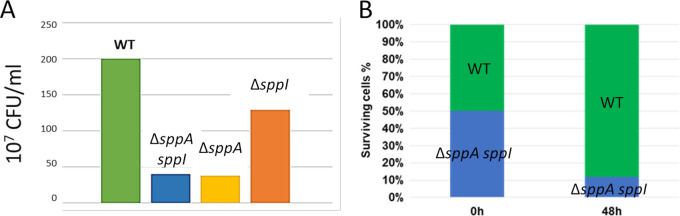
(A) After 48 h of growth in LB medium, cells were plated on LB agar, and the numbers of colonies (CFU per milliliter) were determined for the wild-type (WT) strain, the double-deletion mutant (Δ*sppA sppI*), and the single-deletion strains (Δ*sppA* and Δ*sppI*). (B) Competition experiments were performed by mixing equal numbers of cells of the WT strain and the double-deletion mutant strain (Δ*sppA sppI*). After 48 h of growth in LB medium, cells were plated on LB agar with or without chloramphenicol. The results, expressed as percentages of cells at 0 h and 48 h for each strain (WT and Δ*sppA sppI*), from one representative experiment are shown. This experiment was repeated three times.

While evaluating the secretion ability of the single and double mutant strains, we noticed that, after 48 h, the optical density of the *sppA* mutant culture was lower than that of the wild type (WT) (Fig. 2A). This was also true for the *sppA sppI* double mutant strain, while the effect of the *sppI* deletion was less severe. This suggests a beneficial role of SppA in the long-term survival of B. subtilis cells. To check whether the SppA activity could provide factors beneficial for cell survival, such as amino acids, by releasing them into the growth medium, coculture experiments were performed by mixing wild-type and *sppA* mutant cells in equal amounts and subsequently testing the number of CFU for both strains by plating on lysogeny broth (LB) agar with or without chloramphenicol. The results show that almost no *sppA* cells were able to grow after a 48-h incubation and that the wild-type cells had outcompeted the *sppA* mutant cells upon 48 h of coculturing (Fig. 2B). We conclude from these experiments that (i) SppA provides an important selective advantage to the cells for long-term survival in stationary phase and (ii) wild-type cells are unable to complement or facilitate the survival of the *sppA* mutant cells by releasing particular compounds into the medium. On the contrary, the deletion of *sppI* affected neither the growth rate nor the survival of the *sppI* mutant cells after 48 h.

### SppA promotes resistance to antimicrobial peptides in B. subtilis.

**FIG 3:**
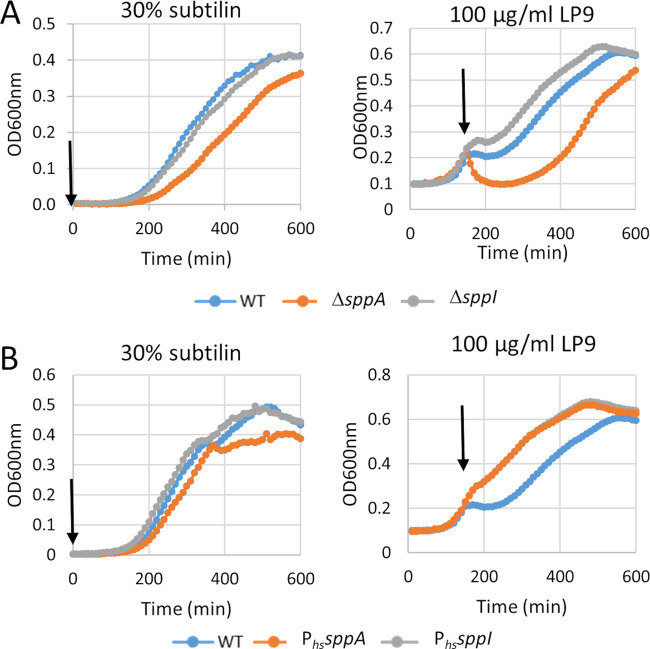
(A) Effects of *sppA* and *sppI* deletion on resistance to subtilin and LP9. For subtilin, cells were inoculated in LB medium supplemented with the indicated concentrations of antimicrobial peptides mentioned above the graphs. For LP9, the antibiotic peptide was added to the medium when cells had reached an OD_600_ of 0.1 at the concentration mentioned above the graphs. (B) Same as described above for panel A. WT, wild-type strain; P*_hs_sppA*, Δ*sppA* strain expressing *sppA* at the *amyE* locus controlled by the IPTG-dependent promoter (P*_hs_*); P*_hs_sppI*, Δ*sppI* strain expressing *sppI* at the *amyE* locus controlled by the IPTG-dependent promoter. Arrows indicate the time at which the antimicrobial peptide was added. Results of experiments with nisin are shown in Fig. S4A in the supplemental material.

SppA was previously demonstrated to be involved in resistance to the lantibiotic nisin (13). Our present results showed that SppA and SppI form a complex in the membrane, and we thus wondered whether SppI plays a role in resistance to antimicrobial peptides. As the *sppA* mutant strain showed altered viability at 48 h, we first checked that none of the *sppA* and/or *sppI* deletion mutants were detectably affected in growth on LB medium or the optical density reached within the first ∼10 h of growth. We next used the lantibiotics subtilin and nisin or the human lysozyme-derived cationic antimicrobial peptide (CAMP) LP9 to test the effect of the deletion of either *sppI* or *sppA* on growth in LB. As shown in Fig. 3A, Fig. S4A, and Fig. S5A, the *sppA-*deficient cells displayed increased sensitivity to all three antibiotic peptides compared to wild-type or *sppI* mutant cells. The *sppI* mutant cells behaved mostly like the wild type, although the *sppI* deletion apparently led to slightly higher resistance to LP9 (Fig. 3A).

10.1128/mSphere.00724-20.5 FIG S4 (A) Effect of different concentrations of nisin and subtilin on the growth of B. subtilis WT (BSB1), Δ*sppA*, and Δ*sppI* strains. (B) Effect on growth of different concentrations of IPTG to express either *sppA* (P*_hs_sppA* strain), *sppI* (P*_hs_sppI* strain), or both (P*_hs_sppA sppI* strain) in the presence of 30% subtilin. Download FIG S4, TIF file, 2.8 MB. Copyright © 2020 Henriques et al. 2020 Henriques et al. This content is distributed under the terms of the Creative Commons Attribution 4.0 International license.

10.1128/mSphere.00724-20.6 FIG S5 (A) Growth of the wild-type strain (BSB1) compared to the deleted strains (Δ*sppA*, Δ*sppI*, and Δ*sppA sppI*) in LB in the presence of increasing concentrations of LP9. The arrows indicate the time when LP9 was added to the culture medium. (B) LP9 resistance of the Δ*sppA*, Δ*sppI*, or Δ*sppA sppI* strain complemented with the corresponding genes at the *amyE* locus under the control of the P*_hs_* promoter inducible with IPTG. Cells were grown in the presence of IPTG, and the arrow indicates when LP9 was added to the growth medium. (C) Effect of erythromycin on the growth of the WT (BSB1), Δ*sppA*, or Δ*sppI* strain. Cells taken during exponential phase were diluted 20 times into LB medium containing erythromycin at the concentration indicated above the graphs. Download FIG S5, TIF file, 2.4 MB. Copyright © 2020 Henriques et al. 2020 Henriques et al. This content is distributed under the terms of the Creative Commons Attribution 4.0 International license.

To check whether the sensitivity of the *sppA* and *sppI* single mutants or the *sppA sppI* double mutant to subtilin or LP9 was directly due to the gene deletions and not to polar effects, we reinserted the *sppA*, *sppI*, or *sppA sppI* genes under the control of the IPTG-inducible P*_hs_* promoter at the *amyE* locus in the corresponding deletion strains. As shown in Fig. 3B and Fig. S5B, in the presence of IPTG, the Δ*sppA* P*_hs_sppA* strain displayed almost wild-type growth in the presence of 30% subtilin, while the resistance of this strain to LP9 appeared even higher than that of the wild-type strain. The overexpression of *sppA* thus provided direct evidence for the involvement of SppA in lantibiotic and LP9 resistance. The level of the lantibiotic resistance was dependent on the IPTG concentration, and the overexpression of the entire *sppA sppI* operon also conferred higher subtilin resistance (Fig. S4B). Of note, the overexpression of *sppI* showed no effect or only slightly elevated antibiotic resistance. Control experiments testing erythromycin, a nonpeptidic antibiotic, were performed. No differences in resistance between the wild type and the deletion mutants were recorded for erythromycin (Fig. S5C). Knowing that SppA is able to degrade fully folded proteins and not only peptides (19), we also tested the effect of chicken egg lysozyme. However, none of the deletion strains showed any differences in growth in the presence of lysozyme, which indicates that SppA cannot degrade lysozyme (data not shown). Altogether, these results show that SppA activity confers resistance to nisin, subtilin, and LP9, whereas SppI might act as a regulator of SppA activity.

### SppI suppresses detrimental effects of SppA protease activity on separation of cells during cell division.

**FIG 4:**
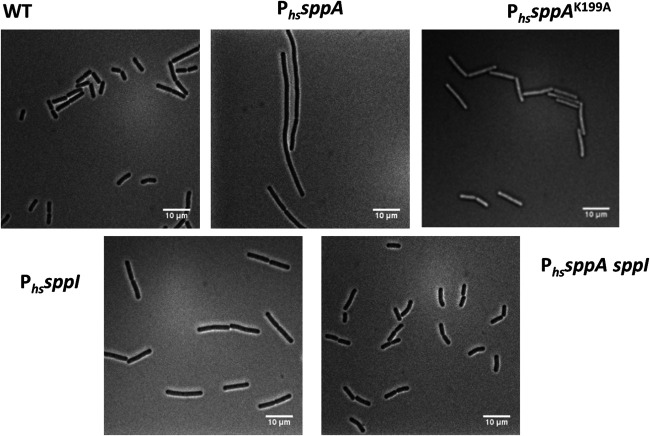
Phase-contrast microscopy images of cells of the wild-type strain, the Δ*sppA* mutant complemented with *sppA* under the control of the P*_hs_* promoter (P*_hs_sppA*), the Δ*sppI* mutant complemented with *sppI* (P*_hs_sppI*), the Δ*sppA sppI* mutant complemented with *sppA sppI* (P*_hs_sppA sppI*), and the Δ*sppA* mutant complemented with the mutated gene *sppA^K199A^* (P*_hs_sppA*^K199A^). The different strains were grown in LB medium supplemented with 50 μM IPTG, and cell samples were taken in the exponential phase of growth for observation.

Although the growth of all the deletion or complementation strains was identical to that of the wild-type strain in LB medium, we checked the cell morphology using a phase-contrast microscope. The Δ*sppA*, Δ*sppI*, and Δ*sppA sppI* cells showed no significant differences in size and general morphology compared to the wild-type cells (data not shown). However, Δ*sppA* P*_hs_sppA* cells grown in the presence of IPTG appeared as long chains of unseparated cells (Fig. 4). On the contrary, the wild-type cells and *sppI* or *sppA sppI* complemented cells showed identical and normal morphologies. Of note, a mix of two morphological states, chains of cells and single or doublet cells, during exponential growth is normally observed for B. subtilis (21). However, all P*_hs_sppA* cells grown in the presence of IPTG showed a homogeneous chained-cell morphology. This is reminiscent of mutants deficient for autolysin activity of Lyt proteins that remodel the cell wall (22). Indeed, 4′,6-diamidino-2-phenylindole (DAPI) staining of the chromosomal DNA revealed that the P*_hs_sppA* cells had problems in separating the two chromosomes between the two daughter cells as DAPI staining was observed across the division septum (Fig. S6). In the P*_hs_sppA* cells, these effects of SppA overexpression on cell morphology were observed with as little as 10 μM IPTG, indicating that even a small increase in SppA expression already altered normal division and separation of the cells. Thus, the protease activity of SppA could act on particular Lyt proteins and/or some of the division proteins localized at the septum. As a control experiment, we checked whether simple steric hindrance in the cytoplasmic membrane due to the expression of SppA might account for this effect. Therefore, instead of expressing the wild-type gene for active SppA from the *amyE* locus, we expressed an *sppA* gene with the K199A active-site mutation from the *amyE* locus, resulting in the production of the proteolytically inactive SppA^K199A^, as shown previously (16). B. subtilis cells expressing SppA^K199A^ showed no difference in size or morphology compared to the wild-type cells (Fig. 4). We therefore conclude that the protease activity of SppA is responsible for the morphological defect of the P*_hs_sppA* cells. Also, the finding that the coexpression of *sppA* and *sppI* under the control of the P*_hs_* promoter (P*_hs_sppA sppI*) did not induce any differences in the cell morphology (Fig. 4) is consistent with the idea that SppI could act as a negative regulator of SppA’s protease activity.

10.1128/mSphere.00724-20.7 FIG S6 Cell morphology of *sppA*-overexpressing strains. All strains were grown in LB until early stationary phase, and a sample was then taken for microscopy analysis. The first panel (left) shows phase-contrast microscopy pictures of uninduced strains. The remaining panels (from left to right) represent the phase-contrast and fluorescence microscopy images of the indicated strains grown in the presence of 50 μM IPTG. The P*_hs_sppA*, P*_hs_sppA sppI*, P*_hs_sppA sppI*^Δ152–164^, and P*_hs_sppA sppI*^Δ129–164^ strains were expressed from a P*_hs_* promoter and induced with 50 μM IPTG. Membranes (falsely colored red) were stained with FM4-64, and nucleoids (falsely colored blue) were stained with DAPI. Bar, 10 μm. Black arrows represent the cell size, and white triangles represent the cell septum or septum formation points. Download FIG S6, TIF file, 2.6 MB. Copyright © 2020 Henriques et al. 2020 Henriques et al. This content is distributed under the terms of the Creative Commons Attribution 4.0 International license.

### Purification of full-length SppA and SppI and the SppA-SppI complex.

**FIG 5:**
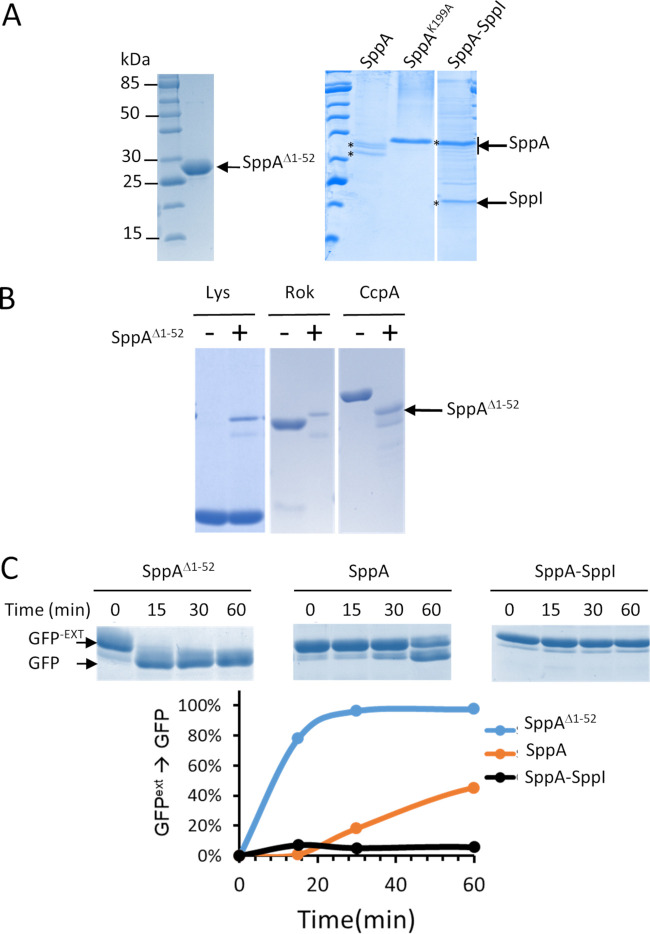
(A) SDS-PAGE analysis of the purified SppA^Δ1–52^, SppA, and SppA^K199A^ proteins and the SppA-SppI complex. All SppA proteins were expressed with a His tag and purified accordingly. SppA^Δ1–52^ was purified from the cytoplasmic fraction of E. coli, while membrane proteins SppA, SppA^K199A^, and SppA-SppI were purified from the cytoplasmic membrane fraction of E. coli. (B) Lysozyme (Lys), B. subtilis Rok, and CcpA were mixed with or without SppA^Δ1–52^, and mixtures were incubated at 37°C for 12 h before being loaded on an SDS-PAGE gel. (C) Protease activity kinetics of SppA^Δ1–52^, SppA, and SppA-SppI mixed with GFP^-ext^ used as the substrate. The mixtures were incubated at 37°C, and at the time indicated, samples were taken and incubated at 95°C for 5 min. All samples were loaded on an SDS-PAGE gel and stained with Coomassie blue. The band intensities were measured using Image Lab (Bio-Rad) and plotted against time.

The full-length B. subtilis *sppA* gene, the mutated *sppA^K199A^* gene version, as well as the whole *sppA sppI* operon were cloned with a 6×His tag sequence fused to the 5′ end of *sppA*, expressed, and purified from E. coli cytoplasmic membranes. Similarly, the *sppI* gene was overexpressed in E. coli, allowing the purification of the His-SppI protein. However, except for full-length SppA^K199A^, the yields were low, and it also appeared that the affinity-purified fractions were contaminated by other E. coli proteins. To improve the purification of the complex, we added a FLAG tag sequence to the 3′ end of *sppI*, allowing the expression of the *6*×*his-sppA sppI-flag* operon and the improved purification of the doubly tagged 6×His-SppA-SppI-FLAG complex by metal affinity chromatography and subsequent anti-FLAG affinity chromatography. This also proved that the B. subtilis SppA and SppI proteins formed a complex in the E. coli membrane. To remove the remaining contaminants, we performed gel filtration, and the resulting purified proteins were analyzed by SDS-PAGE (Fig. 5A). The gel filtration technique also allowed us to estimate the sizes of the native complexes (Fig. S2). Compared to the elution volumes of standard MW markers, the elution volumes indicated apparent MWs of ∼350 kDa for SppA and SppA^K199A^ and ∼160 kDa for His-SppI. Based on these results, we conclude that both the active and inactive SppA as well as SppI were purified as octameric complexes. However, after elution from the gel filtration column, the two proteins of the His-SppA-SppI-FLAG complex appeared in separate fractions, with the elution volumes for each protein corresponding to the MWs of the separated octamers. Thus, although the complex remained stable in the two sequential affinity purifications, it appeared to dissociate during gel filtration.

### Differential sensitivities of full-size proteins to *in vitro* degradation by SppA.

Previous studies have shown that the proteolytic domain of SppA starts at Leu-57 (19). Based on this finding, we cloned and expressed an N-terminally His-tagged truncated version of SppA by removing the first 52 amino acid residues that include the N-terminal transmembrane domain. Although this should lead to a protein of ∼32 kDa, after affinity purification, SppA^Δ1–52^ was detected as a protein of ∼28 kDa (Fig. 5A). This 4-kDa-shorter size actually fits well with the expected size of active SppA, which is known to self-cleave its 4-kDa C-terminal domain to become active (16). We first determined that SppA^Δ1–52^ was active by incubating it with different proteins available in our laboratory. Three cases are exemplified in Fig. 5B: the E. coli protein Rok was completely digested overnight, while the B. subtilis CcpA protein was largely digested, and chicken lysozyme remained intact. After overnight incubation with SppA^Δ1–52^ at 37°C, green fluorescent protein (GFP) or bovine serum albumin (BSA) was also not degraded (data not shown). This shows that some proteins are SppA resistant, presumably due to their structural features.

We also assessed the protease activity of SppA toward a modified GFP, here called GFP^-ext^, which contains a C-terminal extension of 2.8 kDa that is normally used to create a linker for GFP fusion proteins (Fig. S7). After incubation with SppA^Δ1–52^, the size of GFP^-ext^ was reduced by ∼3 kDa and clearly comparable to that of authentic GFP (Fig. 5C), presumably due to degradation of the extension. Since GFP^-ext^ was a convenient substrate for SppA, it was used to monitor the protease activity of different versions of SppA in subsequent experiments.

10.1128/mSphere.00724-20.8 FIG S7 GFP^-ext^. Shown is the sequence of the modified GFP used as the substrate to measure SppA activity, with the GFP sequence itself in red and the C-terminal extension (linker) in black. Download FIG S7, TIF file, 0.1 MB. Copyright © 2020 Henriques et al. 2020 Henriques et al. This content is distributed under the terms of the Creative Commons Attribution 4.0 International license.

### SppI protects SppA from self-cleavage in the SppA-SppI complex.

The SppA protein purified in the presence or absence of SppI was analyzed by SDS-PAGE as shown in Fig. 5A. Only one band was observed for SppI, with an apparent MW of 20 kDa, while at least three major bands potentially corresponding to SppA with apparent MWs ranging from 32 kDa to 34 kDa were detected for SppA purified in isolation. Interestingly, the major SppA band observed in the purified SppA-SppI complex was ∼36 kDa. The inactive SppA^K199A^ also formed a band of ∼36 kDa. We therefore hypothesized that the 36-kDa band of SppA derived from the purified SppA-SppI complex corresponded to full-length SppA, including its C-terminal domain, while the 32-kDa band from the separately purified SppA corresponded to SppA lacking the C-terminal domain. To validate this hypothesis, the SppA and SppI bands marked with stars in Fig. 5A were excised from the gel and analyzed by MS. This showed that SppI was indeed the major protein in the 20-kDa band and that SppA was the major protein in the three other bands. Importantly, for SppA purified in the SppA-SppI complex, two peptides, ^310^SEIDFLNMR^317^ and ^318^EILSQSGSPR^327^, belonging to the C-terminal domain of SppA, were detected in the 36-kDa band. According to a previous study (19), these two peptides are not present in the active form of SppA. When SppA was separately purified, these two peptides were not detected in the 32-kDa band. We therefore conclude that SppA in the SppA-SppI complex still contains most of its C terminus. In turn, this suggests that SppA is present in an inactive state in the SppA-SppI complex, where SppI could protect SppA from self-cleavage. On the contrary, when separately purified, SppA could be in the active form without its C-terminal domain. Therefore, we tested the protease activities of SppA, SppA in the SppA-SppI complex, and the truncated form of SppA, SppA^Δ1–52^, using GFP^-ext^ as a substrate. The SppA quantity for each form of SppA was estimated from band intensities after SDS-PAGE and then adjusted in the mixtures with the GFP^-ext^ substrate. Upon incubation at 37°C, samples were taken at different intervals and analyzed by SDS-PAGE. The appearance of the product, i.e., GFP without the extension, was used as a measure of SppA activity (Fig. 5C). The results showed that SppA^Δ1–52^ was the most active form, while SppA purified separately was also able to degrade the linker extension of the GFP^-ext^ substrate albeit with low efficiency. In contrast, SppA in the SppA-SppI complex was unable to degrade the linker extension of GFP^-ext^, as no product was detectable during the time course of the experiments (60 min). After overnight incubation, the GFP product was detectable, indicating that SppA in the SppA-SppI complex became active after a long period of incubation (data not shown). Based on these observations, we conclude that in the SppA-SppI complex, SppA is held inactive by SppI, presumably because SppI prevents self-cleavage of the C-terminal domain.

### SppA activity is regulated by the C-terminal domain of SppI.

In an attempt to further decipher how SppI maintains SppA in its inactive form, we took advantage of the cell chaining phenotype observed when SppA is overexpressed by itself from the *amyE* locus (Fig. 4). This phenotype is correlated with SppA activity, as the expression of the inactive SppA^K199A^ led to a wild-type cell phenotype. Conversely, the coexpression of SppA and SppI induced no change compared to the wild-type phenotype, indicating that SppA was not active and therefore negatively regulated by SppI. To investigate which part of SppI might be involved in the control of SppA activity, we used a dissection approach by deleting parts of the C terminus of SppI. Structural predictions for SppI obtained with various programs (HMMTOP, JUFO, DAS-TMfilter, TMpred, and TMHMM) indicated the presence of two or three transmembrane helices with a C-terminal domain facing either the cytoplasm or the cell wall (Fig. S9). While the first two transmembrane helices were consistently predicted, the prediction of the third transmembrane helix was inconclusive. The last residue of the possible third helix was predicted to be Phe-140, leaving a 25-amino-acid-long C-terminal domain for SppI. To assess the function of this domain, two strains were constructed, which ectopically express either P*_hs_sppA sppI*^Δ152–164^, i.e., SppI truncated for its last 12 residues, or P*_hs_sppA sppI*^Δ129–164^, i.e., SppI truncated for its last predicted helix, from the *amyE* locus. Cells of these two strains were grown in LB medium until reaching exponential phase, and their morphology was inspected by phase-contrast microscopy (Fig. S6). This showed that the cell chaining phenotype became detectable when the last 12 amino acids of SppI had been removed, and it was much stronger for the strain expressing the shortest version of SppI that lacks the third predicted helix. Since SppA activity is responsible for the cell chaining phenotype, as shown in Fig. 4, we conclude that the C terminus of SppI is required to control the proteolytic activity of SppA.

## DISCUSSION

### Role of SppA.

It was previously reported that the deletion of *sppA* had an effect, although quite mild, on the maturation rate of the overexpressed secretory protein AmyQ (11), and this effect was also reported more recently for a B. licheniformis *sppA*-deficient strain, which displayed a general defect in protein secretion (20). B. subtilis SppA shows 49% identical residues and conservative replacements with the N-terminal domain of the E. coli signal peptide peptidase SppA (11), implying that SppA might act as a signal peptide peptidase in B. subtilis. However, in 2011, RasP, an S2P protease that cleaves peptides within the plane of the membrane, was proposed to be the major signal peptide peptidase in B. subtilis (10). A major role of RasP in protein secretion was later confirmed by the overexpression of RasP in B. subtilis, which increased the yield of secreted proteins up to 10-fold (23). Our present results show that the final levels of the overexpressed secretory protein AmyM in the *sppA* deletion mutant were not significantly lower than those observed for the WT strain. Likewise, SppA was not important for α-amylase or protease secretion under fermentation-mimicking conditions (24). Conversely, in the *sppA*-overexpressing strain, the levels of AmyM were negatively affected. These findings are different from the results reported for B. licheniformis where the overexpression of SppA was apparently beneficial for the secretion levels of nattokinase and AmyL (20). Species-specific differences can be the reason for these opposite observations, but they may also be explained by different experimental conditions or differences in the level or timing of overexpression of *sppA* due to the use of different promoters in different studies. Nonetheless, based on previous and present observations, we conclude that the role of SppA in protein secretion in B. subtilis is limited. Instead, the present study shows that SppA is involved in resistance to nisin, subtilin, and LP9, which is in agreement with previous observations that showed that *sppA*, a member of the σ^W^ regulon, is involved in lantibiotic resistance (13). This view is further supported by our observation that after incubation of the purified SppA^Δ1–52^ in subtilin-containing LB medium for 12 h at 37°C, the *sppA* mutant and wild-type cells grew identically in this medium, indicating that the subtilin had been degraded (Fig. S8).

10.1128/mSphere.00724-20.9 FIG S8 Growth of the wild-type (BSB1) and Δ*sppA* strains in the presence of subtilin. 0% subtilin, LB without subtilin; 30% subtilin, LB supplemented with 30% subtilin; 30% subtilin (16 h at 37°C), LB supplemented with 30% subtilin and left for 16 h at 37°C before inoculation; 30% subtilin SppA^Δ1–52^ or proteinase K, subtilin left overnight in the presence of one of the proteases in a reaction buffer (20 mM Tris-HCl [pH 7.5], 200 mM NaCl) before being added to LB. Download FIG S8, TIF file, 0.3 MB. Copyright © 2020 Henriques et al. 2020 Henriques et al. This content is distributed under the terms of the Creative Commons Attribution 4.0 International license.

10.1128/mSphere.00724-20.10 FIG S9 Membrane topology predictions for SppI. The number of predicted transmembrane helices (TMHs) varied from 2 TMHs (TMHMM and Protter) to 3 TMHs (Phyre2 and psipred). TMpred gives the third TM helix (from positions 115 to 139) a low probability score compared to the two first TMHs of SppI. The location outside or inside the cytoplasm of the C-terminal domain of SppI is therefore unknown. Download FIG S9, TIF file, 0.4 MB. Copyright © 2020 Henriques et al. 2020 Henriques et al. This content is distributed under the terms of the Creative Commons Attribution 4.0 International license.

### The SppA-SppI complex.

The two genes *sppA* and *sppI* are organized in an operon under the control of the alternative sigma factor σ^W^ (13, 25) and possibly the housekeeping sigma factor σ^A^ (14). The operon structure of these two genes, *sppA* and *sppI*, is well conserved in bacilli and related bacteria like the pathogen Listeria monocytogenes. In this report, we show that SppA and SppI form a complex in the B. subtilis membrane. The protease SppA was purified from the E. coli membrane fraction with an apparent molecular weight of an octamer, in accordance with the crystal structure of SppA truncated from its N-terminal transmembrane domain, SppA^Δ2–54^ (19). Its partner protein SppI was also purified with the apparent molecular weight of an octamer. Although the two proteins were purified as a complex by sequential affinity purification with two different tags, subsequent gel filtration led to the complete dissociation of the two proteins, suggesting a loose interaction between the two octamers. This interaction between multimers of different proteins is reminiscent of the membrane protein complexes formed by the protease FtsH and HflK/HflC (26). FtsH is a hexamer with most of its protease domain facing the cytoplasm. HflK and HflC, two membrane proteins that associate first as heterodimers, interact with E. coli FtsH to form a huge complex of 1 MDa, the FtsH holoenzyme. It was shown that HflKC inhibits the protease activity of FtsH for one of its substrates, the membrane protein SecY (27).

### The protease activity of SppA is regulated by SppI.

**FIG 6:**
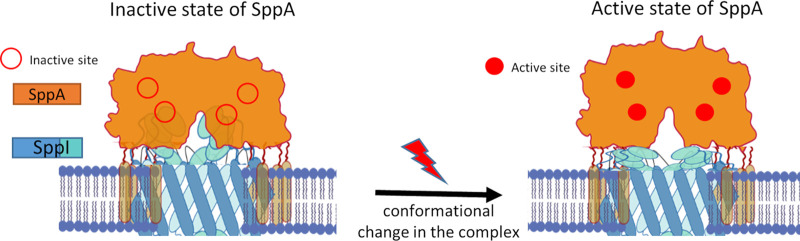
Model for the activation of SppA in the SppA-SppI complex. In the complex formed by SppA and SppI, SppA was found to be inactive due to the presence of SppI, whose C-terminal domain appears to be important for controlling SppA activity. The active sites of SppA are formed by amino acid residues belonging to two adjacent monomers of SppA and therefore require a close interaction between two monomers of the SppA octamer (19). SppA inhibition could be due to steric interference of SppI between the SppA monomers, which would drive both parts of the active sites apart. In response to a signal of an unknown nature, a conformational change in SppI would activate the protease activity of SppA by allowing the active sites of SppA to be reconstituted.

*In vitro*, we showed that, when in complex with SppI, the protease activity of SppA is very low compared to that of SppA alone. *In vivo*, we showed that the activity of overexpressed SppA caused morphological defects in the cells, whereas the cell morphology remained unchanged when SppI was coexpressed with SppA. On this basis, we conclude that SppI negatively controls SppA activity. *In vivo*, we further showed that the last 25 amino acids of the C-terminal domain of SppI are involved in this inhibitory mechanism and that removing the last 45 amino acids of SppI completely abolishes SppA regulation. Nam and Paetzel (16), who solved the structure of the inactive form of B. subtilis SppA with its C-terminal region bound to the active site, previously proposed that SppA processes its C terminus to become active. Thus, the SppA C terminus would act as a negative regulator domain, presumably to prevent SppA from becoming active too early during protein translocation. Interestingly, our MS analysis identified two peptides of the C-terminal domain of SppA but only when SppA was coexpressed and purified as a complex with SppI. Therefore, it appears that SppA is unable to self-process its C terminus in the presence of SppI and that SppA in the SppA-SppI complex is maintained in an inactive form by SppI. In contrast, Nam and Paetzel (16) noticed that the self-cleavage of the C terminus of SppA^Δ2–54^, which lacks the N-terminal transmembrane domain, was very fast and complete in E. coli. Noticeably, B. subtilis SppA, purified without SppI, invariably showed multiple bands on SDS-PAGE gels that differed in size by 2 to 4 kDa, with the highest-MW band corresponding to the band of SppA in the SppA-SppI complex (Fig. 5A). This suggests that SppA was able to cleave its C terminus, but only partially, and that this process was slow under the tested conditions. This could relate to the N-terminal transmembrane domain of SppA because the protease activity of full-size SppA was lower than that of the soluble SppA^Δ1–52^ form. Interestingly, E. coli FtsH has been shown to self-cleave the last 7 amino acids of its C-terminal domain *in vivo*, and the deletion of *hflKC* retarded this C-terminal self-cleavage of FtsH (28). Conversely, in the case of B. subtilis SppA, SppI inhibits SppA by preventing it from self-cleaving its own C terminus, presumably until physiological harm caused by antibiotic peptides requires the activation of SppA. In this scenario, SppA would be activated only when necessary since its activity could be detrimental to the cell if not sufficiently controlled. At this stage, the inhibitory mechanism by which the C-terminal domain of SppI carries out its control over SppA is unknown. A propeptide blocking the protease active site is a classical mechanism, as exemplified for the subtilisin AprE (29). By analogy, SppI could negatively control the self-cleavage of SppA’s C-terminal “propeptide,” either by direct interaction with this propeptide and maintaining it inside the catalytic groove or by slightly altering the overall SppA octameric structure, which could lead to a conformational change in the active site (Fig. 6). As opposed to HflKC, which modulates FtsH in E. coli most probably by preventing substrate access to FtsH, SppI seems to act only as an inhibitor that prevents the C-terminal domain self-cleavage and autoactivation of SppA. Detailed studies on specific substrates would be needed to rule out a more complex role for SppI, although it is difficult to envisage how SppA could be modulated and inhibited by SppI once SppA’s C-terminal domain is cleaved.

### SppI: sensor and activator?

Finally, our present findings raise questions about the nature of the signal that triggers the activation of SppA and how SppI inhibition is relieved. For example, the PDZ domain of E. coli DegS acts as an inhibitory domain for DegS until it binds hydrophobic peptides of the C terminus of misfolded proteins leading to the release of the inhibition (30). Similar to the PDZ domain of DegS, SppI could be viewed as a sensor of antibiotic peptides or an unfolded part of substrate proteins, and the C-terminal domain of SppI could act as the inhibitory/activating domain for SppA. Interestingly, SppI belongs to the RDD protein superfamily, which is characterized by the presence of three highly conserved residues, arginine-aspartate-aspartate. Three other proteins in B. subtilis (YczC, YckC, and YxaI) belong to this protein family. In fact, the Pfam database indicates that this RDD domain is present in many transmembrane proteins, mostly in *Bacteria* but also with some occurrences in *Archaea* and *Eukaryota*. Until recently, the molecular function of this RDD domain was completely elusive. However, looking for novel Na^+^/H^+^ antiporters, Shao et al. identified a protein named RDD in the moderate halophile Halobacillus andaensis. The RDD protein functions as a Na^+^ (Li^+^, K^+^)/H^+^ antiporter, and 4 out of 6 conserved arginine or aspartate residues were indispensable for this antiporter activity (31). Although B. subtilis SppI did not appear in the sequence alignment shown by Shao et al., it is clearly related to H. andaensis RDD, and the corresponding *rdd* gene is even adjacent to a putative *sppA* gene. Moreover, B. subtilis SppI shares all 6 conserved arginine and aspartate residues identified in the homologs of *H. andaensis* RDD, and overall, the two protein sequences show 39% identity. We have therefore checked whether the deletion or overexpression of *sppI* could alter or improve the NaCl or alkaline pH resistance of B. subtilis. In both cases, the mutant strains appeared as resistant as the wild-type strain, with no effect on the growth rate or level of produced biomass in the stationary phase of growth. Furthermore, we tested the overexpression of *sppI* in an *mprA* deletion mutant, as the *mprA* mutation causes high NaCl sensitivity. However, no improvement in NaCl resistance was observed. We can thus conclude that SppI does not contribute strongly to the general resistance to salt or alkalinity stresses in B. subtilis. However, we also cannot rule out the possibility that SppI acts as a cation antiporter in B. subtilis as in *H. andaensis*. For instance, SppI could still act as a sensor of cellular cationic concentration changes. In the case of salt stress and/or leakage of cations induced by lantibiotic peptides, which are believed to create holes in the membrane, SppI could undergo a conformational change that in turn would relieve the inhibitory effect of SppI’s C-terminal domain on SppA. Hence, the SppA-SppI complex may represent a two-protein system dedicated to fighting the effects of antimicrobial peptides, where SppI could sense the damage caused and activate SppA by relieving its inhibition. Clearly, additional experimental evidence that B. subtilis SppI acts as a cation antiporter will be needed to further establish this intriguing model, which implicates SppA-SppI complexes in resistance to antimicrobial peptides. This is important as the ability of cationic antimicrobial peptides (CAMPs) to synergistically enhance the efficacy of antibiotics has been documented (32, 33). A possible bacterial defense system against CAMPs was recently described, which involved the enzymatic neutralization of LP9 by a secreted peptidylarginine deiminase in Porphyromonas gingivalis (34). However, the resulting citrullination of LP9 was proven not to be the only mechanism to provide resistance to this CAMP (34). As such, gingipains, which are secreted proteases of P. gingivalis, were previously shown to degrade the human CAMP hBD3, suggesting a role in resistance to CAMPs (35). By analogy, the B. subtilis membrane-bound protease SppA, in complex with and controlled by SppI, might be considered a member of this first line of defense of the Gram-positive bacterium B. subtilis. Hence, as the *sppA sppI* operon is widely present in the *Bacilli* class and in some pathogenic Gram-positive bacteria such as Listeria monocytogenes, a role in evading host innate immunity should also be investigated in these species.

## MATERIALS AND METHODS

### Bacterial strains.

For all experiments with B. subtilis, we used the strain BSB1, a Trp^+^ derivative of the 168 wild-type strain described previously (14). PCRs used the chromosomal DNA of BSB1 as the template. We used E. coli MC1061 for all intermediate cloning and E. coli ER2566 for the overexpression of SppA and SppI.

### Deletion of *sppA*, *sppI*, or the *sppA sppI* operon.

The *sppA* and/or *sppI* mutant strains were obtained by sequence replacement of the genes with a chloramphenicol resistance (Cm^r^) cassette. PCR fragments of approximately 1 kb were obtained by amplifying the upstream and downstream regions of *sppA* and/or *sppI* using the appropriate pairs of primers, KO-P1/KO-P2 and KO-P5/KO-P6, respectively (data not shown). Next, the Cm^r^ resistance cassette, approximately 1 kb, was PCR amplified from a pUC19 plasmid. Overlapping ends of the fragments, which were included in the primer sequences, allowed the joining of the three PCR fragments by overlap PCR. The final PCR product was used to transform competent cells of B. subtilis BSB1. The deletion of genes of interest from the native locus was confirmed by PCR amplification and sequencing.

### Tagging of SppA and SppI.

The pMUTIN-SPA vector was used as previously described (36) to construct B. subtilis BSB1-derivative strains expressing SppI-SPA or SppA-SPA fusion proteins in which the SPA tag is fused to the C terminus of the protein. Sequencing from the chromosomal DNA of each strain was performed to check the DNA sequences encoding the fusion regions. Cell extracts were used for Western blotting and immunodetection with anti-FLAG antibodies (Sigma-Aldrich) to check the expression of the fusion proteins. As SppA-SPA was not detectable, we constructed another strain harboring an additional copy of the whole *sppA sppI* operon modified as *6×his-sppA-sppI-SPA.* This construct was placed under the control of the isopropyl β-d-1-thiogalactopyranoside (IPTG)-dependent P_hyperspank_ (P*_hs_*) promoter and inserted at the *amyE* locus using the pDR111 plasmid. The resulting strain expressed the two proteins with different tags, His-tagged SppA and SppI-SPA.

### Overexpression of SppA and SppI or their truncated versions in B. subtilis.

To create B. subtilis strains overexpressing SppA and/or SppI, the P*_hs_* promoter was fused to the respective gene sequences. The PCR-amplified genes were ligated to the pDR111 plasmid and subsequently used to transform E. coli MC1061 competent cells. After sequencing, plasmids that allow the integration of the cloned fragment into the *amyE* locus were used to transform B. subtilis BSB1 competent cells. The transformants were selected on lysogeny broth (LB) agar plates supplemented with spectinomycin. The insertions in the *amyE* locus were checked by the starch-iodine test and further confirmed by PCR amplification of the region and DNA sequencing.

### Overexpression of SppA and SppI in E. coli.

To obtain His-tagged versions of SppA (full-length protein or a soluble truncated version), SppI, or the SppA-SppI complex, we used a modified pJ411 plasmid (DNA 2.0) allowing the ligation of NdeI-XhoI-digested DNA fragments downstream of the T7 promoter. DNA fragments of *sppA*, *sppI*, or *sppA-sppI* were obtained by PCR amplification from B. subtilis BSB1 chromosomal DNA with specific primers encoding 6×His at the 5′ ends as well as NdeI and XhoI restriction sites. To obtain a DNA fragment corresponding to *sppA*^Δ1–52^, a specific primer was designed to hybridize after the first 156 nucleotides of the *sppA* gene. All PCR fragments were digested with NdeI and XhoI and ligated to the pJ411 plasmid, and the ligation mixtures were used to transform E. coli MC1061. The generated plasmids were verified by sequencing and used to transform the E. coli ER2566 expression strain.

### Purification of the SPA-tagged membrane protein SppI.

For SPA tag purification of the membrane protein SppI-SPA, we used a method described previously by Charbonnier et al. (17), a modified method of that described previously by Delumeau et al. (15), as the protocol had to be adapted for membrane proteins. Briefly, two affinity chromatography steps were applied using calmodulin (CaM) resin (calmodulin Sepharose 4B; GE Healthcare) followed by anti-FLAG antibody resin (anti-FLAG M2 affinity gel; Sigma). The *sppI-spa* strain was inoculated into a 5-liter flask containing 2 liters of LB medium supplemented with 0.6 mg/ml erythromycin and 1 mM IPTG, and cells were grown at 37°C with vigorous shaking (200 rpm/min). Cells were collected by centrifugation at 6,000 × *g* for 10 min when the optical density at 600 nm (OD_600_) reached ∼1. Cell pellets were washed by resuspension in 40 ml of cold buffer A (10 mM Tris-Cl [pH 7.5], 150 mM NaCl) before being centrifuged again as described above and stored at −80°C until use. Cell pellets were resuspended in 20 ml of buffer A supplemented with 1 mg/ml lysozyme, 1 mM EDTA, and 5 μl of Benzonase (Invitrogen) and incubated in a water bath at 37°C for 15 to 30 min. To remove the cell debris, the sample was centrifuged at 4,000 × *g* for 30 min at 4°C. The membrane fraction was obtained after ultracentrifugation at 100,000 × *g* for 1 h at 4°C. The membrane pellet was resuspended using buffer A supplemented with 1% *n*-dodecyl β-d-maltoside (DDM) and 3 mM CaCl_2_, 200 μl of preequilibrated CaM resin was added, and the mixture was placed on a rotating wheel for at least 2 h at 4°C. The whole mixture was transferred into a disposable column, the resin was washed with buffer A supplemented with 1% DDM and 0.1 mM CaCl_2_, and elution was achieved by adding 0.75 ml of buffer A supplemented with 1% DDM and 3 mM EGTA. The eluate was transferred into a new disposable column and mixed with 200 μl of anti-FLAG resin. After 2 h, the resin was washed with 2 ml of buffer A supplemented with 1% DDM, and elution was achieved by adding 1 ml of 100 mM glycine (pH 3). To concentrate the proteins, they were precipitated with 10% trichloroacetic acid (TCA) before being analyzed by SDS-PAGE and identified by mass spectrometry (MS).

### Protein identification by mass spectrometry.

To identify the proteins copurified with SppI-SPA, we used the same method as the one described previously (15) except that the samples were first separated by a short run of SDS-PAGE, and pieces of gel containing the proteins were cut out to be analyzed by MS.

### Purification of 6×His-SppA and 6×His-SppA-SppI complexes.

The E. coli strains expressing N-terminally 6×His-tagged SppA, N-terminally 6×His-tagged SppI, N-terminally 6×His-tagged SppA-SppI, or N-terminally 6×His-tagged SppA^Δ1–52^ were cultured in 2 liters of LB medium supplemented with 30 μg/ml kanamycin and grown until the OD_600_ reached 0.8 at 37°C. The expression of tagged proteins was induced by the addition of 250 μM IPTG to the culture medium, and the cultures were left to grow overnight at 16°C. After centrifugation, cell pellets were resuspended in 40 ml of a solution containing 20 mM Tris-HCl (pH 7.5) and 250 mM NaCl, lysed by sonication, and centrifuged at 4,500 rpm for 30 min to remove the cell debris. The membrane and cytosolic fractions were separated by ultracentrifugation at 100,000 × *g* for 1 h at 4°C. The membrane fractions were solubilized with the same buffer supplemented with 1% DDM and then loaded onto a Ni^2+^ affinity column (Ni-NTA agarose; Qiagen) preequilibrated in the same buffer. The Ni-NTA columns were washed with 50 ml of a solution containing 20 mM Tris-HCl (pH 7.5), 250 mM NaCl, and 0.01% DDM supplemented with 5 mM imidazole and then with 20 ml of the same buffer supplemented with 20 mM imidazole. The proteins bound to the Ni-NTA resin were then eluted with 4 ml of 200 mM imidazole. The same protocol was used to purify 6×His-tagged SppA^Δ1–52^ from the cytosolic fraction of the E. coli cells and without the addition of DDM in the buffers. The His-tagged protein-containing fractions were concentrated by using Amicon Ultra-15 centrifugal filters. The 6×His-tagged proteins purified from E. coli membranes, i.e., 6×His-SppA, 6×His-SppI, and 6×His-SppA-SppI, were further purified by gel filtration using a Superdex 200-GL 10/300 column (GE Healthcare). After analysis by SDS-PAGE, the purest gel filtration fractions containing the proteins of interest were pooled and concentrated by using Amicon Ultra-15 centrifugal filters.

### BN-PAGE and LC-MS analysis.

The membrane protein complexes of a B. subtilis membrane fraction prepared as described above were separated by blue native electrophoresis. The electrophoresis lane was cut in 10 equidistant pieces. The proteins present in each gel band were identified by LC-MS to determine the migration position of SppA and SppI. The blue native PAGE and mass spectrometry procedure details are included in supplemental Text S1.

10.1128/mSphere.00724-20.1 TEXT S1 Supplemental materials and methods. Download Text S1, DOCX file, 0.01 MB. Copyright © 2020 Henriques et al. 2020 Henriques et al. This content is distributed under the terms of the Creative Commons Attribution 4.0 International license.

### Pulse-chase protein labeling.

Pulse-chase labeling of B. subtilis was performed with 25 μCi Easy tag [^35^S]methionine (PerkinElmer) as previously described (23). Subsequently, ^35^S-labeled AmyQ was immunoprecipitated with protein A affinity medium (Mabselect Sure; GE Healthcare Life Sciences) and specific polyclonal antibodies. Precursor and mature forms of the immunoprecipitated labeled AmyQ were separated by lithium dodecyl sulfate-PAGE using 10% NuPage gels (Life Technologies) and visualized using a Cyclone Plus PhosphorImager (PerkinElmer) as previously described (23).

### Growth inhibition assays.

B. subtilis strains were grown overnight at 37°C in LB. The cultures were then diluted to optical densities at 600 nm of 0.01 for growth inhibition studies with nisin or subtilin or 0.1 for growth inhibition studies with LP9. Aliquots of 100 μl of these suspensions were pipetted into the wells of a 96-well plate. Bacteria were then grown with vigorous shaking at 37°C in a BioTek Synergy 2 microplate reader (BioTek Instruments Inc., Winooski, VT, USA). Nisin, subtilin, or LP9 was added at different concentrations and at different time points, after which bacterial growth was monitored for approximately 10 h.

### Microscopy.

For phase-contrast and epifluorescence microscopic observations, samples of cells were loaded on a thin layer of 1% agarose poured on microscope slides (Gene Frame; Thermo Scientific). Images of the cells were obtained using a Leica DMRA2 instrument with a 100× objective. Cell membranes were visualized after incubation with FM4‐64 (1 μg/ml).
