# Supplementary figures and images for: SppI Forms a Membrane Protein Complex with SppA and Inhibits Its Protease Activity in Bacillus subtilis
Source: mSphere. 2020 Oct 7;5(5):e00724-20. doi: 10.1128/mSphere.00724-20 (PMC7568657; doi:10.1128/mSphere.00724-20)

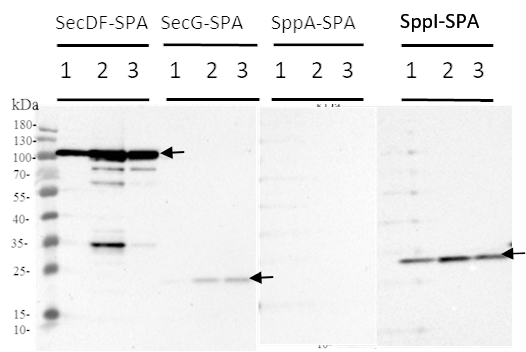

Supplement: FIG S1 [file mSphere.00724-20-sf001.tif]

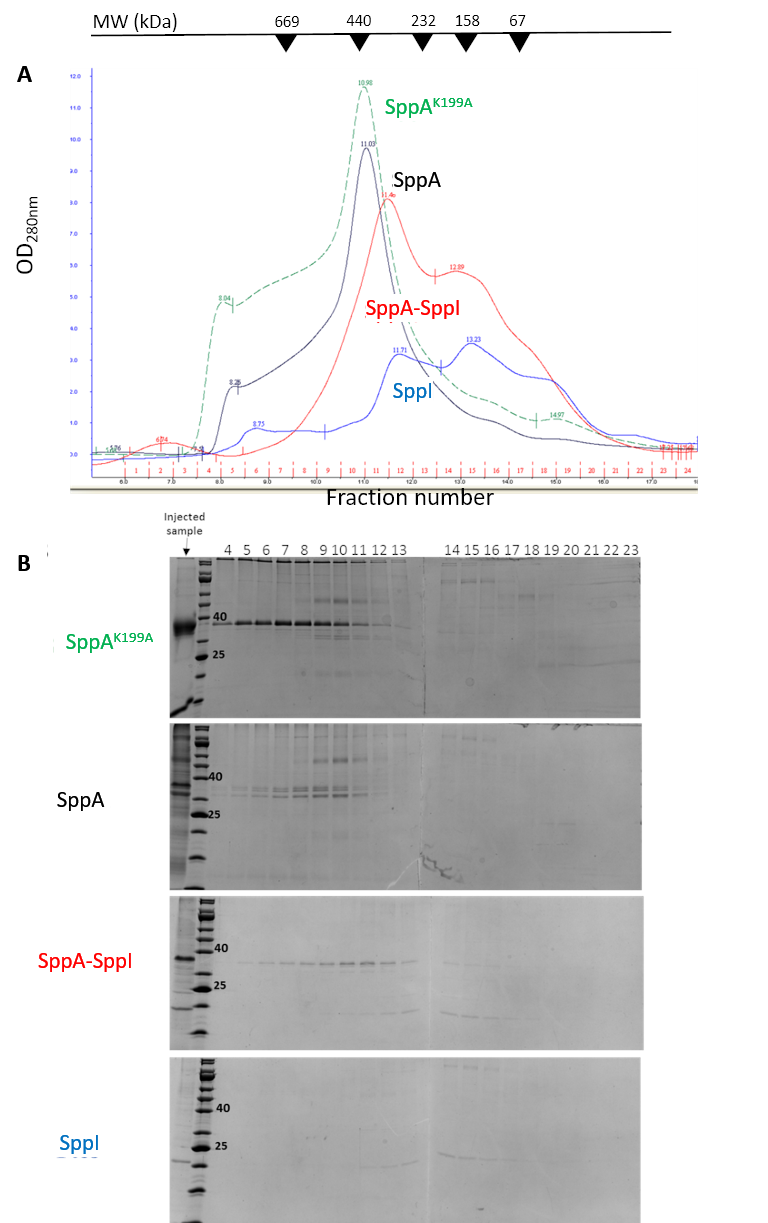

Supplement: FIG S2 [file mSphere.00724-20-sf002.tif]

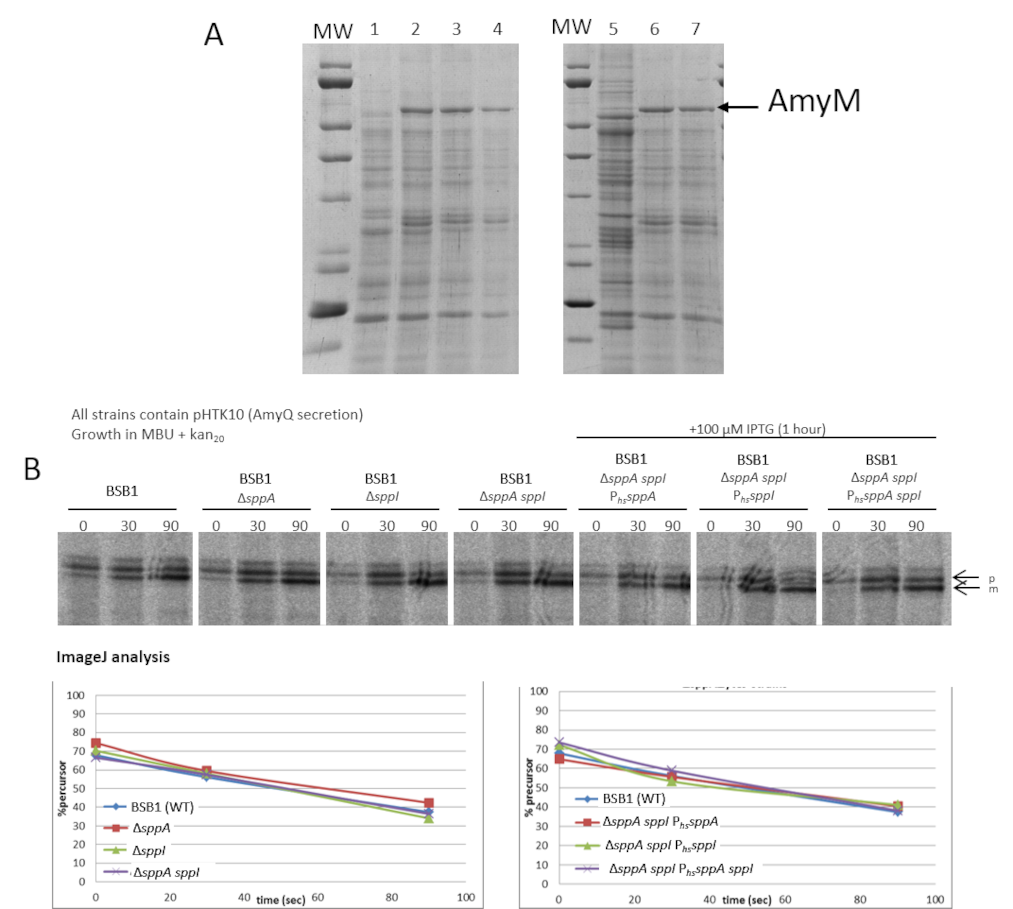

Supplement: FIG S3 [file mSphere.00724-20-sf003.tif]

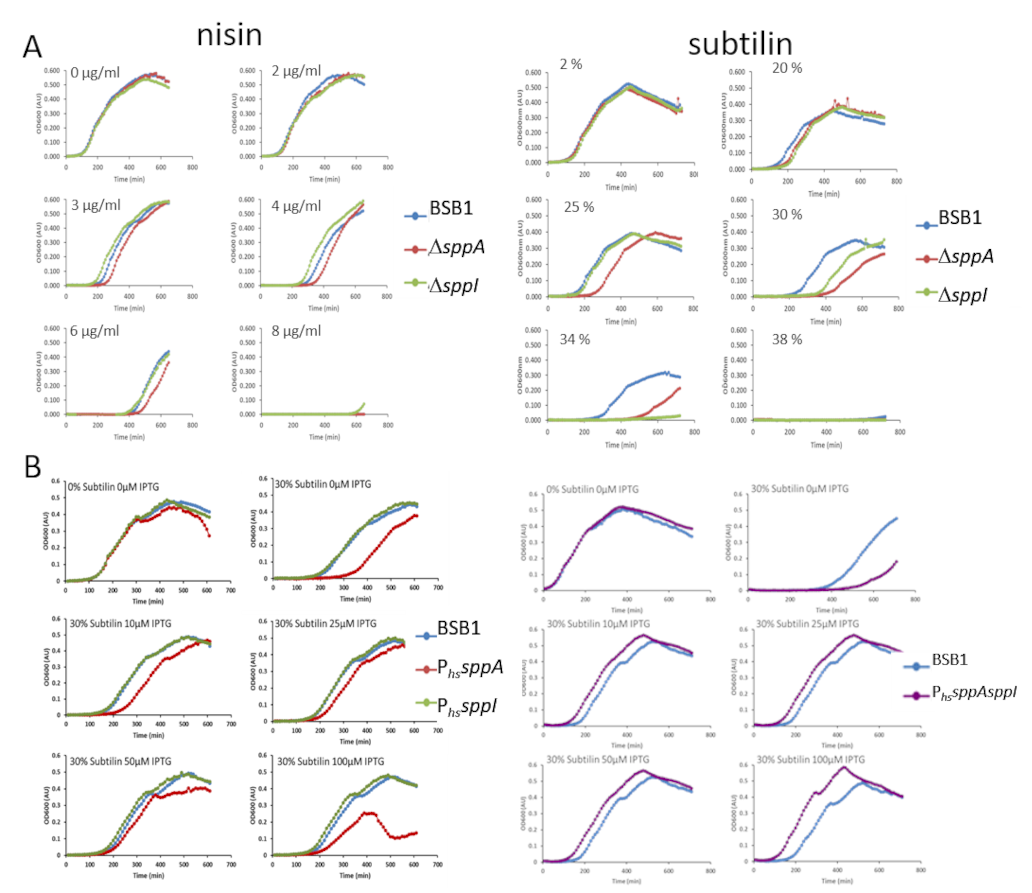

Supplement: FIG S4 [file mSphere.00724-20-sf004.tif]

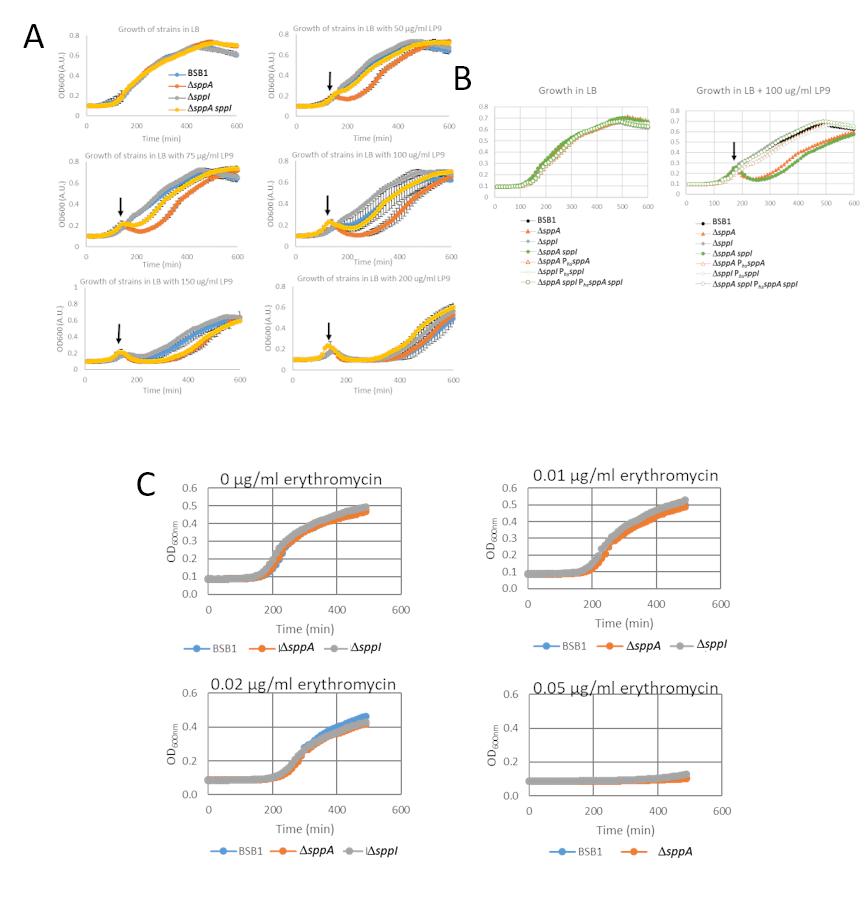

Supplement: FIG S5 [file mSphere.00724-20-sf005.tif]

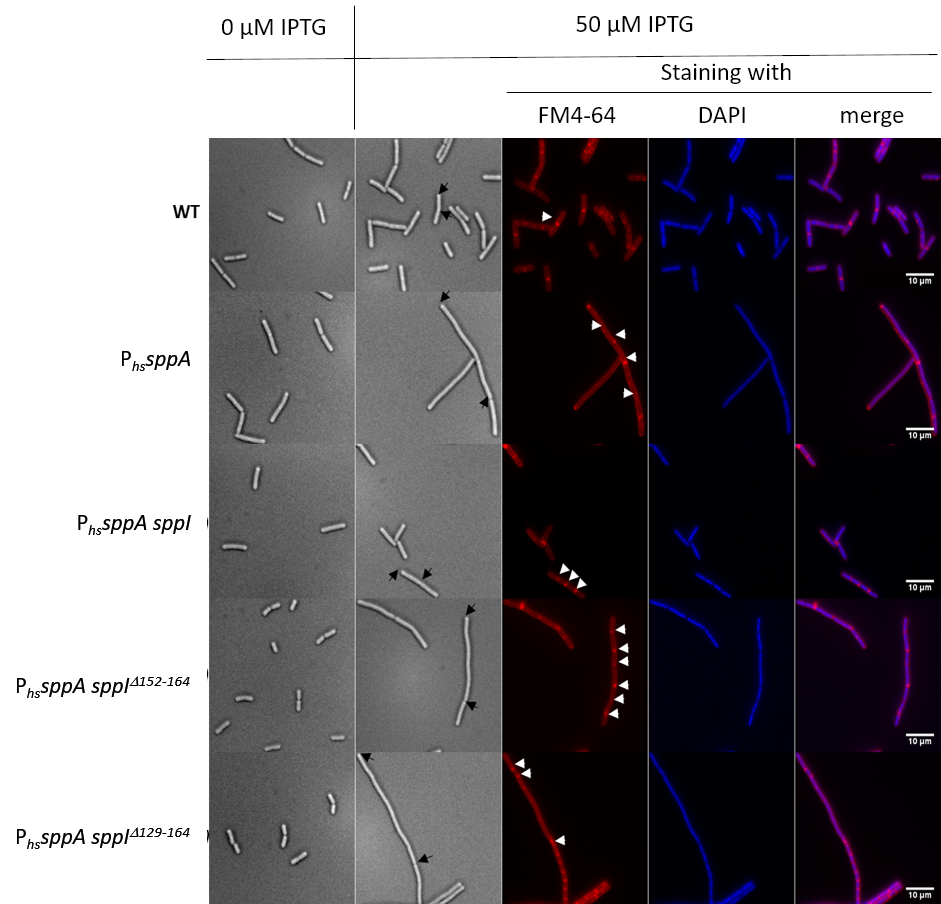

Supplement: FIG S6 [file mSphere.00724-20-sf006.tif]

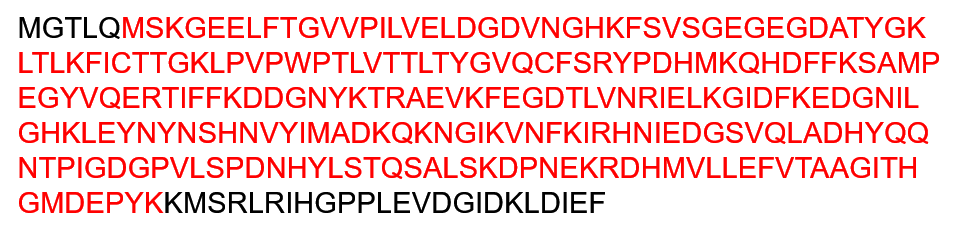

Supplement: FIG S7 [file mSphere.00724-20-sf007.tif]

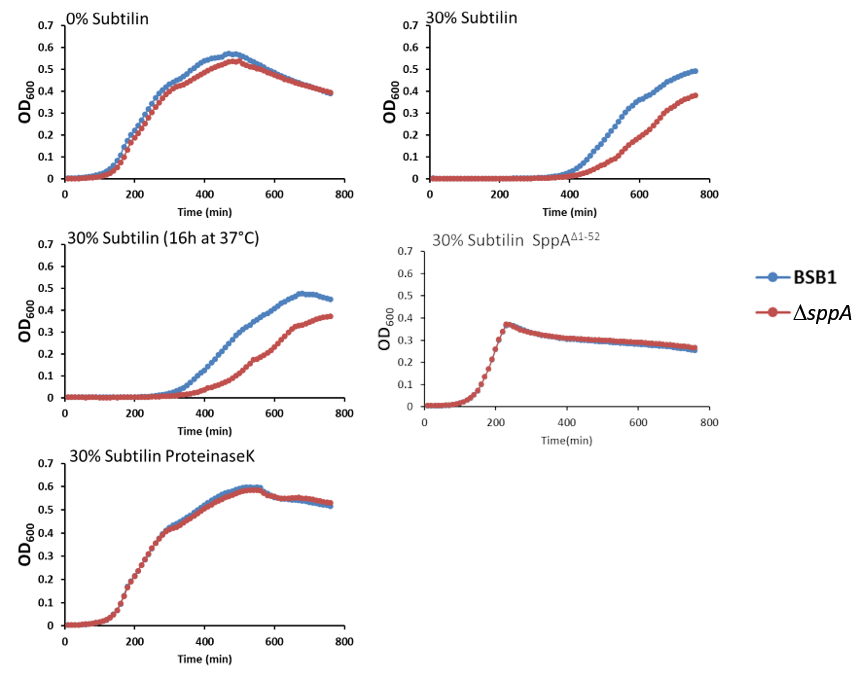

Supplement: FIG S8 [file mSphere.00724-20-sf008.tif]

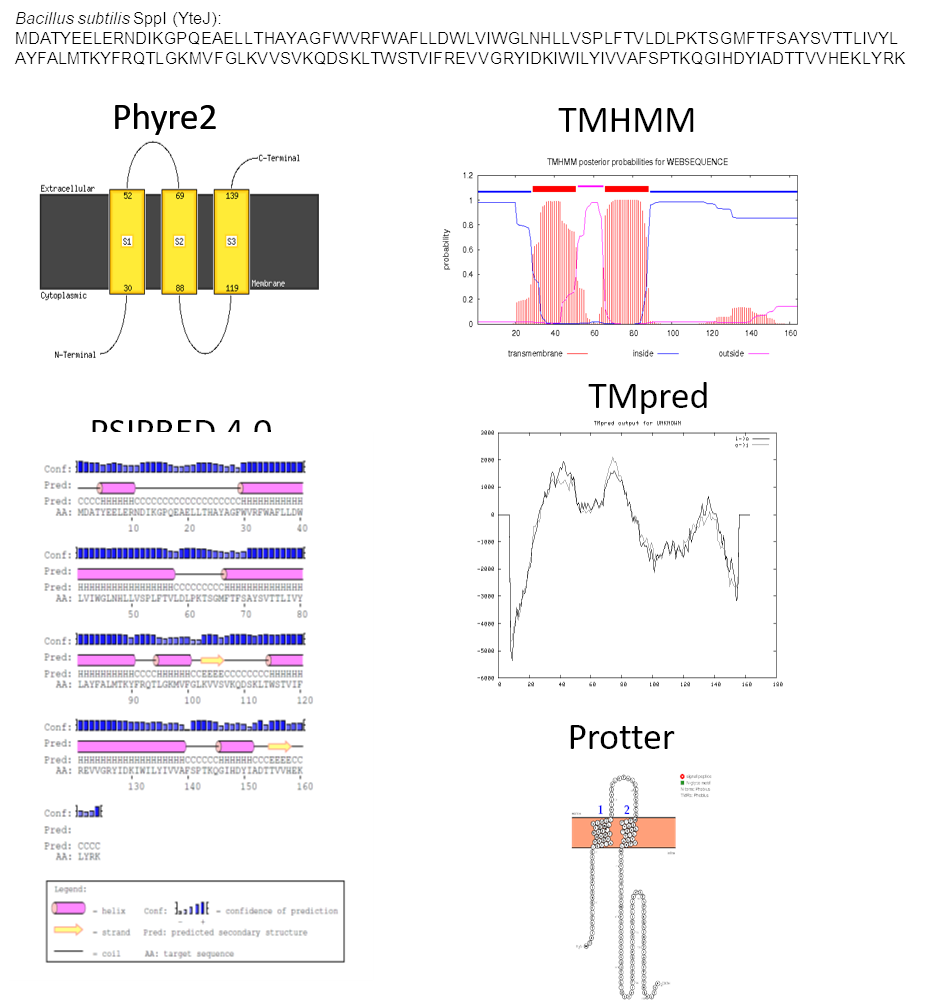

Supplement: FIG S9 [file mSphere.00724-20-sf009.tif]
