# Supplementary material for: SppI Forms a Membrane Protein Complex with SppA and Inhibits Its Protease Activity in Bacillus subtilis
Source: mSphere. 2020 Oct 7;5(5):e00724-20. doi: 10.1128/mSphere.00724-20 (PMC7568657; doi:10.1128/mSphere.00724-20)
Supplement: TEXT S1 [file mSphere.00724-20-s0001.docx]

**Supplementary Data**

**SppI forms a membrane protein complex with SppA and inhibits its protease activity in *Bacillus subtilis***

Gabriela Henriques^1^, Stephen Mc Govern^1^, Jolanda Neef^2^, Minia Antelo-Varela^3^, Friedrich Götz^4^, Andreas Otto^3^, Dörte Becher^3^, Jan Maarten van Dijl^2^, Matthieu Jules^1^ and Olivier Delumeau^1^#.

**Material and Methods**

**Blue-Native electrophoresis:**

For Blue-Native electrophoresis, cells grown in LB were harvested at OD_600nm_ 0.5 by centrifugation at 6000 rpm for 10 min at 4°C. The pellet was washed with 40 ml of cold resuspension buffer A (10 mM Tris-Cl pH 7.5, 150 mM NaCl), centrifuged at 4500 rpm for 10 min at 4°C and instantly frozen in liquid nitrogen. The cells were resuspended in 20 ml of buffer A supplemented with 1 mg/ml of lysozyme, 1 mM EDTA and 5 µl of Benzonase (Invitrogen), and incubated in a water bath at 37°C for 15-30 min. To remove the cell debris the samples were centrifuged at 4500rpm for 30 min at 4°C and the pellet was discarded. Membrane and cytosolic fractions were then separated by ultracentrifugation at 100,000 *g* for 1 h at 4°C. The membrane protein samples were solubilized in the same buffer supplemented with 1% n-Dodecyl β-D-maltoside (DDM), mixed with NativePAGE sample buffer and with NativePAGE 5% G-250 Sample Additive (Invitrogen), and incubated for 1h on ice. The proteins were then separated by electrophoresis using NativePAGE 3%–12% Bis-Tris gels (Invitrogen) with NativePAGE running buffer and NativePAGE Dark Blue Cathode Buffer (Invitrogen) for 20 to 30 min at 150 V; the latter buffer was then substituted with NativePAGE Light Blue Cathode Buffer (Invitrogen) for 60 min at 150V. The electrophoresis tank was maintained on ice throughout the procedure. The gel was stained by Blue Safe Protein Stain (BioRad).

**Analysis by LC-MS/MS**

Each lane was cut into ten equidistant pieces that were further treated to be analysed by liquid chromatography LC-MS/MS analysis according to (Bonn et al., 2014). Briefly, after staining, the gel pieces were washed twice with water to remove excessive Coomassie stain. All gel pieces were further cut into small cubes and transferred into a low binding tube. The gel pieces were washed at least three times for 15 min with 750 μL of washing buffer (0.2 M ammonium bicarbonate in 30% (v/v) acetonitrile) at 37°C under vigorous shaking. The destained gel pieces were dried in a vacuum centrifuge at 30°C and rehydrated with 2 μg/mL trypsin solution (Promega) for 15 min. Excessive trypsin solution was removed, and the digest was performed overnight at 37°C. The gel pieces were covered with water, and the peptides were eluted from the gel matrix by immersion of the reaction tube in an ultrasonic bath for 15 min. The supernatant containing the peptides was removed, transferred to a glass vial, and dried in a vacuum centrifuge and resuspended in 10 µL of MS-grade water. For LC−MS/MS analyses of gel samples, in-house self-packed columns were prepared and used with an EASY-nLC II system (Thermo). The peptides were loaded onto the column by the LC system with 10 μL of buffer A (0.1% (v/v) acetic acid) at a constant flow rate of 500 nL/min without trapping. The peptides were subsequently eluted using a nonlinear 100 min gradient from 1 to 99% buffer B (0.1% (v/v) acetic acid in acetonitrile) with a constant flow rate of 300 nL/min and injected online into the mass spectrometer. MS and MS/MS data were acquired with a LTQ Orbitrap (Thermo). After a survey scan at a resolution of 30,000 in the Orbitrap with activated lockmass correction, the five most abundant precursor ions were selected for fragmentation. Singly charged ions as well as ions without detected charge states were not selected for MS/MS analysis. Collision-induced dissociation (CID) fragmentation was performed for 30 ms with normalized collision energy of 35, and the fragment ions were recorded in the linear ion trap. Database searching was done with Sorcerer-SEQUEST 4 (SageN). After extraction from the raw files, *.dta files were searched with Sequest against a target−decoy database with a set of common laboratory contaminants. The target database was the Uniprot reference database of *B. subtilis* 168 (downloaded May 21, 2014). The resulting *.out files were compiled with Scaffold 4. Proteins were only considered as identified if at least two unique peptides matching solid quality criteria (delta cN > 0.1 and XCorr > 2.2; 3.3; 3.75 for doubly, triply, or higher charged peptides) had been assigned, resulting in a false positive rate (FPR) below 0.1% on protein level.
